# Service Users’ Experiences of a Nationwide Digital Type 2 Diabetes Self-Management Intervention (Healthy Living): Qualitative Interview Study

**DOI:** 10.2196/56276

**Published:** 2024-07-18

**Authors:** Rhiannon E Hawkes, Jack S Benton, Sarah Cotterill, Caroline Sanders, David P French

**Affiliations:** 1 Manchester Centre for Health Psychology, Division of Psychology and Mental Health School of Health Sciences University of Manchester Manchester United Kingdom; 2 Division of Population Health, Health Services Research & Primary Care School of Health Sciences University of Manchester Manchester United Kingdom

**Keywords:** type 2 diabetes, digital interventions, behavior change, self-management, implementation, qualitative methods

## Abstract

**Background:**

Diabetes Self-Management Education and Support programs for people living with type 2 diabetes mellitus (T2DM) can increase glycemic control and reduce the risk of developing T2DM-related complications. However, the recorded uptake of these programs is low. Digital self-management interventions have the potential to overcome barriers associated with attendance at face-to-face sessions. *Healthy Living* is an evidence-based digital self-management intervention for people living with T2DM, based on the Healthy Living for People with Type 2 Diabetes (*HeLP-Diabetes*) intervention, which demonstrated effectiveness in a randomized controlled trial. NHS England has commissioned Healthy Living for national rollout into routine care. Healthy Living consists of web-based structured education and *Tools* components to help service users self-manage their condition, including setting goals. However, key changes were implemented during the national rollout that contrasted with the trial, including a lack of facilitated access from a health care professional and the omission of a moderated online support forum.

**Objective:**

This qualitative study aims to explore service users’ experiences of using Healthy Living early in the national rollout.

**Methods:**

A total of 19 participants were interviewed via telephone or a videoconferencing platform. Topics included users’ experiences and views of website components, their understanding of the intervention content, and the overall acceptability of Healthy Living. Transcripts were analyzed thematically using a framework approach.

**Results:**

Participants valued having trustworthy information that was easily accessible. The emotional management content resonated with the participants, prompting some to book an appointment with their general practitioners to discuss low mood. After completing the structured education, participants might have been encouraged to continue using the website if there was more interactivity (1) between the website and other resources and devices they were using for self-management, (2) with health professionals and services, and (3) with other people living with T2DM. There was consensus that the website was particularly useful for people who had been newly diagnosed with T2DM.

**Conclusions:**

Digital Diabetes Self-Management Education and Support programs offering emotional aspects of self-management are addressing an unmet need. Primary care practices could consider offering Healthy Living to people as soon as they are diagnosed with T2DM. Participants suggested ways in which Healthy Living could increase interaction with the website to promote continued long-term use.

## Introduction

### Background

People living with type 2 diabetes mellitus (T2DM) are at risk of developing a range of health complications, including loss of vision, nerve pain, limb amputation, and cardiovascular problems [1]. However, many of these complications can be prevented when individuals self-manage their condition effectively. Diabetes Self-Management Education and Support (DSMES) programs can provide information to guide behavior changes such as improving diet and increasing physical activity to support blood glucose control and learning to cope with negative emotions [2,3]. Systematic reviews have shown that DSMES programs improve service users’ clinical and psychosocial outcomes (eg, improved glycemic management and improved diabetes knowledge) and reduce health care costs [3,4]. Therefore, DSMES programs are now recommended by the National Institute of Health and Care Excellence for all people diagnosed with T2DM [1].

DSMES programs are typically delivered via face-to-face group sessions (eg, Diabetes Education and Self-Management for Ongoing and Newly Diagnosed [5] and X-PERT Health [6] in the United Kingdom). However, recorded attendance at these face-to-face programs remains low globally [2]. For example, in the United States, only 6.8% of the people who were newly diagnosed with T2DM and held private health insurance attended a DSMES session within the first 12 months of diagnosis [7]. Figures are comparable in the United Kingdom, with only 7% of newly diagnosed patients with T2DM recorded as attending a session within their first year of diagnosis [8]. Further research in the United Kingdom has shown that younger people were less likely to attend a 9-month face-to-face behavior change program targeting the prevention of T2DM [9,10]. Digital interventions have the potential to address logistical challenges that attending face-to-face sessions might pose (eg, scheduling, travel, work, and childcare) [11], providing an alternative for those who do not want to attend group sessions [12], and thus may meet the needs of younger people. Therefore, NHS England has recently committed to expanding T2DM support through digital technologies and self-management programs [13].

**Figure 1:**
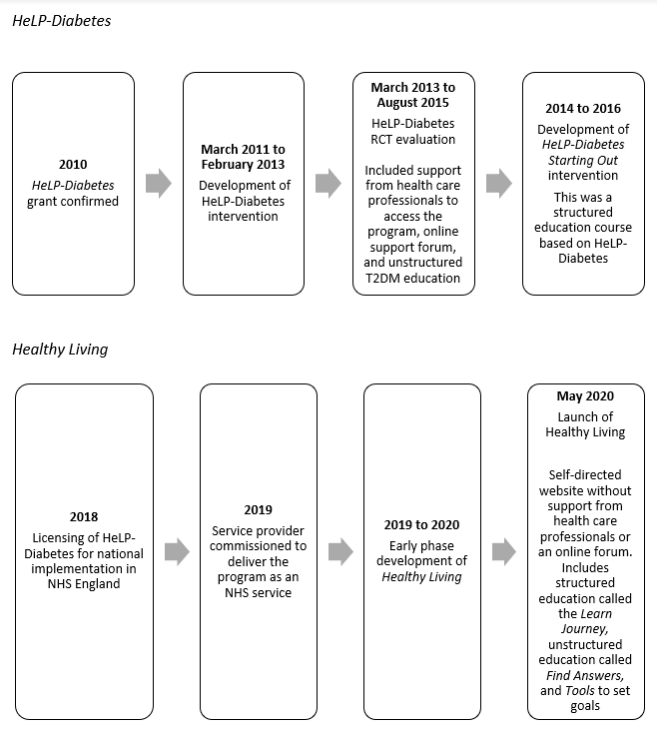
Timeline of intervention development since 2010. HeLP-Diabetes: Healthy Living for People with type 2 diabetes; NHS: National Health Service; RCT: randomized controlled trial; T2DM: type 2 diabetes mellitus.

A digital intervention designed to provide ongoing self-management support for people living with T2DM was Healthy Living for People with Type 2 Diabetes (*HeLP-Diabetes*). A randomized controlled trial (RCT) of HeLP-Diabetes found that the digital program was feasible to deliver, was acceptable to service users, reduced blood glucose, and was cost-effective for the National Health Service (NHS) [14]. HeLP-Diabetes was an unstructured program, which provided access to educational content without following a linear pathway [15]. Following this RCT, the researchers developed an additional structured educational component called *Help-Diabetes: Starting Out*, based on the content in the original HeLP-Diabetes website [16]. This additional development was due to changes in NHS policy in 2013, which stipulated that self-management programs were only eligible for accreditation if they followed a structured pathway with a clear curriculum (Figure 1).

In 2019, NHS England commissioned a national rollout of a version of HeLP-Diabetes into routine health care [17] (Figure 1 [18]). The program is called *Healthy Living*, which has been developed and delivered by an external digital service provider as an NHS service [19] and includes the structured education component developed after the RCT. Access to Healthy Living is currently by self-referral and general practitioner (GP) referral, and it is a self-contained, self-directed service. Healthy Living is a web-based program that includes a structured education pathway (*Learn journey*) and a *Tools* section where service users can set goals; self-monitor their health (eg, diet, steps, weight, and blood glucose levels); and find answers to specific questions. The program includes behavior change techniques (the “active ingredients” of interventions to produce behavior change) [20] and self-management content based on those that were originally included in the HeLP-Diabetes intervention. The self-management content was guided by the Corbin and Strauss [21] model, which includes 3 types of tasks: medical management (eg, adopting healthy behaviors and taking medicines); emotional management (eg, managing emotions including anger, guilt, shame, and despair); and role management (eg, managing changes in relationships, work patterns, and day-to-day activities). Multimedia Appendix 1 [14,16,18,22-36] provides further information on the development and content of Healthy Living, including screenshots of website content. A detailed description of the behavior change content in Healthy Living is described elsewhere [18].

Previous research from this program of work has assessed the extent to which the content of Healthy Living retained fidelity to the intervention content of the original HeLP-Diabetes RCT and identified reasons for changes implemented in the national rollout of Healthy Living [18]. This assessment found that Healthy Living had good fidelity to the behavior change techniques and self-management content of the HeLP-Diabetes RCT. However, there were key changes implemented during the national rollout that contrasted with the RCT, comprising (1) the inclusion of a structured web-based learning curriculum due to changes in the NHS policy, (2) a lack of facilitated access to the program from a health care professional due to fewer resources in general practice, and (3) the omission of a moderated online support forum due to low uptake in the HeLP-Diabetes RCT [14].

Given that Healthy Living has been rolled out nationally across England, it is important to understand how Healthy Living is experienced by service users and the extent to which the program is acceptable for people using the service. Previous qualitative research has investigated participant experiences of using the original HeLP-Diabetes intervention in the RCT. For example, participants who used HeLP-Diabetes reported feeling better informed and more aware of their T2DM self-management, and they valued the support they received from the program, including having access to health care professionals [37]. Further qualitative research on the structured education component, *HeLP-Diabetes: Starting Out*, suggested that the course was acceptable to service users, although completion rates were low, some of whom attributed this to competing priorities such as work and family responsibilities [16]. However, research is yet to obtain the views from service users who have not taken part in a trial, and we are yet to understand whether any of the changes in the current nationally implemented version of HeLP-Diabetes (eg, reduced interaction with health care professionals and other patients living with T2DM) have implications for service user experience. Furthermore, it is important to assess how the intervention content is understood, as this will impact program engagement and outcomes.

### Objectives

This study aimed to explore service users’ experiences of using Healthy Living. Specific objectives were to (1) understand the extent to which the different components of Healthy Living were acceptable to service users; (2) understand the contents of Healthy Living that the service users engaged with; (3) understand any barriers to engagement with, and use of, Healthy Living; and (4) investigate how the Healthy Living intervention material is understood (“intervention receipt”) and how this impacts the use of intervention materials (“intervention enactment”) [38].

## Methods

Methods are reported in accordance with the Standards for Reporting Qualitative Research [39] (Multimedia Appendix 2).

### Design

This study used a cross-sectional design, where semistructured qualitative interviews asked service users for their views about using Healthy Living.

### Participants

Participants were people living with T2DM who had actively engaged with Healthy Living within the last 12 months (completed at least 30% of the structured education or set a goal using the Tools) to assess how the program was experienced by users and to ensure participants were able to answer the interview questions with sufficient detail.

### Sampling and Procedures

The service provider delivering the intervention sent emails to cohorts of service users inviting them to complete a web-based screening survey (via REDCap [Research Electronic Data Capture]; Vanderbilt University) [40], which asked service users to fill out a demographic questionnaire and register their interest in taking part in an interview (Multimedia Appendix 3). Of those service users who registered their interest, a member of the research team (JSB) purposively sampled a selection of service users (aiming to achieve demographic diversity in age, gender, ethnicity, socioeconomic status, and time since T2DM diagnosis) to invite them to take part in an interview, before requesting the service provider to send the next batch of recruitment emails. This enabled us to review the demographic groups of users who had already been recruited and revise the email strategy accordingly (Multimedia Appendix 4). The selected service users were emailed an invitation to take part in an interview along with an information sheet. The recruitment strategy (eg, the wording of the recruitment email from the service provider and contents of the REDCap questionnaire) was discussed and refined with members of a Patient and Public Involvement and Engagement (PPIE) group before commencing recruitment. Of the 29 participants who were contacted to take part, 8 (28%) refused to participate (no response after contact: 3/8, 38%; participants felt they were unsuitable candidates for the interview: 3/8, 38%; participants did not remember signing up via the REDCap survey: 1/8, 12%; participants wanted a face-to-face interview: 1/8, 12%). A further 2 (7%) of the 29 participants took part in an interview, but it was apparent that they had taken part in a different program, so they were excluded from the final analysis.

One-to-one semistructured interviews were conducted by a male researcher (JSB; research associate) who had a PhD and training in qualitative methods. JSB described the aim of the study to participants as wanting to understand their experiences of using Healthy Living. Participants were interviewed via either telephone or a videoconferencing platform (Zoom; Zoom Video Communications, Inc), and complete informed consent was audio recorded before the interview. There were no individuals present during the interviews other than the researcher (in a private office) and the participant. Each interview was recorded using an encrypted audio recorder, transcribed verbatim, and pseudonymized for analysis. Interviews lasted between 30 and 60 minutes. Recruitment was stopped when it appeared to the researchers (JSB and DPF) that no new content was being discussed in the final 2 interviews.

### Materials

A topic guide was used to organize the semistructured interviews, with open-ended questions and additional probes (Multimedia Appendix 5). Questions were asked in line with the objectives, including service users’ experiences of using Healthy Living and its components. Field notes were made following each interview.

### Researcher Positioning

The researcher who conducted the interviews (JSB) had a background in health psychology and thus had a strong understanding of the type of behavior change support delivered to people living with long-term health conditions. This may have influenced some of the questions asked in the interviews (eg, with more focus on the individuals rather than wider socioeconomic status constraints). The lead author who analyzed the data (REH) also had a background in health psychology, with >5 years of experience working in diabetes prevention and self-management research.

The wider team (SC, CS, and DPF) has extensive experience conducting independent evaluations of large-scale behavior change programs, including T2DM projects. No members of the research team are currently living with T2DM. We worked closely with a PPIE group (n=8; female: n=5, 62%; male: n=3, 38%; all who were at risk of or living with T2DM). The PPIE group advised the research team on all patient-facing materials, including the wording of the interview schedule, and they advised on the recruitment strategy before data collection. They also provided feedback and interpretations of the findings during the analysis stages, including a discussion on the importance of interactive digital technology and the emotional management aspects of living with T2DM, which was incorporated into the final analysis.

### Analysis

As we wanted to understand participants’ views and experiences of specific features of the intervention that had been adapted for the national program implementation, we analyzed the data thematically and organized them using a framework approach [41]; this involved the development of a framework matrix that allowed for the comparison of findings across participants on key issues where relevant. Data were analyzed from a realist perspective, which assumes that the language used directly reflects participants’ perception of their reality.

A coding framework was developed based on what had changed from the original HeLP-Diabetes RCT [18] and the National Institutes of Health Behavior Change Consortium framework to assess intervention receipt [38]. This informed the development of a priori thematic codes (eg, “understanding of self-management content,” “enactment of educational content,” and “online forum”). Additional codes were also developed inductively during data analysis to capture nuances in the data (eg, “support sought as a result of the program”). This approach allowed us to answer the specific research questions while allowing important insights to be produced inductively. Transcripts were coded to items in the coding framework (Multimedia Appendix 6) and then charted into a framework matrix by 1 researcher (REH), where a succinct description of what was coded for each item of the framework was summarized for each participant. This allowed for the comparison of findings across participant cases. Data were discussed among the authors to identify themes relevant to the research questions, with illustrative extracts and interpretive themes refined through discussion at regular analysis meetings. NVivo software (version 12; Lumivero) was used to facilitate the coding and analysis of the data.

### Ethical Considerations

This study was reviewed and approved by the Yorkshire and the Humber–Leeds West NHS Research Ethics Committee (20/YH/0250). Interview data were deidentified during transcription. All participants provided complete informed consent before the interview. As a "thank you" for taking part in this research, participants could opt to receive £50 (US $65) compensation (either via a voucher or bank transfer).

## Results

### Overview

The 19 interviewees comprised almost even numbers of male (n=10, 53%) and female (n=9, 47%) participants and had a median age of 61 (IQR 53-73, range 43-81) years. The sample had little ethnic diversity but a good spread in terms of deprivation (Table 1). A total of 12 (63%) interviews took place via telephone and 7 (37%) took place via Zoom between October and December 2021.

**Table 1:** Participant characteristics (N=19).

Characteristic				Values
Age (y), median (IQR)				61 (53-73)
**Sex, n (%)**				
	Female		9 (47)	
	Male		10 (53)	
**Ethnicity, n (%)**				
	Black British		1 (5)	
	White British		18 (95)	
**IMD^a^ score, n (%)**				
	1 (least deprived)		2 (10.5)	
	2		6 (32)	
	3		5 (26)	
	4		4 (21)	
	5 (most deprived)		2 (10.5)	
**Time since diagnosis (y)**				
	Values, median (range)		5 (11 months-29 years)	
	**Values, n (%)**			
		0-1	3 (16)	
		1-2	4 (21)	
		2-5	3 (16)	
		5-10	3 (16)	
		>10	6 (32)	

^a^IMD: Index of Multiple Deprivation scores associated with the lower super output area derived from venue postcodes, ranging from the most deprived areas in England to the least deprived areas in England [42].

A total of 4 themes were generated from the analysis: information is there at the touch of a button; improved emotional management; experiences of structured education; and the importance of technological, professional, and social interactivity (Multimedia Appendix 7).

### Theme 1: Information Is There at the Touch of a Button

Healthy Living was valued by participants, as it provided them with a trusted source of information that was “there at the touch of a button” (P6). The NHS branding of the website was perceived as crucial (P6, P10, P13, P16, and P17), especially by participants who had received conflicting information from other sources in the past (P13). Participants contrasted this to websites such as Facebook, which was described as a less-trusted source of information (P10 and P16). A participant stated as follows:

I’m not interested in treating myself as a Guinea pig, I want something which is the proper facts. And because this says NHS, I believe they’re going to be proper facts.

P13, male participant aged 65 years

Most participants (15/19, 79%) reported learning something new from the educational content of Healthy Living, including clarifying things regarding their medical management that were previously misunderstood (P14) and increasing the awareness of how to self-manage their condition (P1 and P19):

Well I think it’s helped me realise that there is hope obviously outside of just avoiding sugar, on the diabetes front. There are lots of other aspects to healthy living that need to be maintained and used.

P1, male participant aged 74 years

Even participants who had been diagnosed with T2DM for a long time reported to have learned something new from reading the educational content (P1, P4, P10, P13, and P14):

It would be the learning goals definitely, because there was stuff, even though I am ten plus years diagnosed there was still bits I didn’t know. And obviously there’s scope there to put new research in, so it’s got a really good potential place ahead.

P10, female participant aged 43 years

Participants placed value on continually learning and updating their knowledge on how to manage their T2DM. Some participants (8/19, 42%) expressed a desire to know more about the dietary aspects of their self-management, including what foods they should and should not be eating (P5), recipes (P3, P7, P8, P9, and P16), substitutes for sugary food (P5 and P16), the amount of sugar (P12) and carbohydrates (P7) in different foods, and how specific foods impacted on blood glucose levels (P3 and P18). Others wanted information on actions to take if blood sugar levels became too high (P10) and the methods to bring blood sugar levels under control (P13). However, some participants (4/19, 21%) reported already knowing most of the information presented in the educational content and consequently felt they had not learned anything new from engaging with the website (P4, P12, and P18). This caused some to disengage (P4 and P5). A participant stated as follows:

I think if you don’t know anything it’s probably useful but I already...I’ve had numerous health problems so I have reasonable knowledge of useful information and some information about diabetes along with that. It was probably a bit condescending if you already know all of this stuff but good if you don’t know anything.

P12, female participant aged 57 years

Therefore, while most participants found some information on Healthy Living useful, some participants (2/19, 11%) who had been diagnosed with T2DM for a long time felt that this program was particularly suited to those who were newly diagnosed (P4 and P18), with newly diagnosed participants reporting that Healthy Living would have been more beneficial if it was offered straight away after their diagnosis (P2 and P12).

### Theme 2: Improved Emotional Management

Participants had expected Healthy Living to include information on ways to manage their diet and medications, but many had not expected to see information on emotional management. This was a welcome addition to the website for most participants, as they had not been told about the emotional impact of T2DM previously and had not encountered it on other self-management programs they had attended (P4, P10, P16, and P19):

I thought it was interesting that it wasn’t just about what the causes are and how you should control your sugars, but things like emotional impact, just general well-being impact.

P14, male participant aged 69 years

...[E]veryone has told me what diabetes was going to do me physically but no one had said anything about mentally. So, that’s the site that I learnt more about it, no one had mentioned that at a doctor’s appointment, no one had mentioned it at the nurse’s appointments, it was the first place it had even been mentioned to me and all of a sudden, I thought I’ve got that problem and now I know, is this the reason why I’m feeling like that. It hadn’t been mentioned anywhere else, I hadn’t learned about it from a book or anywhere else and I remember reading it and deciding that from being not happy in life a little bit, thinking, well this must be what the problem is, thinking hilariously I felt a little bit better, thinking this might be what the problem is and then I spoke to my nurse in the doctor’s surgery.

P16, male participant aged 45 years

Many participants (12/19, 63%) reported to find the emotional management content useful (P2, P4, P6, P8, P9, P10, P11, P13, P14, P16, and P17), increasing their understanding of the link between low moods and T2DM (P6). Even those who had not experienced low mood appreciated receiving informational support about this aspect of their self-management (P11, P14, and P15). Reading the emotional management content prompted some participants to book an appointment with their GP or nurse to discuss their low moods (P4, P6, and P16), thus suggesting that participants felt comfortable to subsequently discuss aspects of emotional management with health care professionals. This emotional management content seemed to be legitimizing the experiences of participants and reassured them that their experiences of low mood could be explained, despite the self-led nature of the program with no interaction from others:

Yeah, yes, it was, it was, it was kind of reassuring you that it wasn’t just something that you were going through, it was linked with your diabetes, and it’s very common as well and I think that was reassuring, to learn that it wasn’t just me, that it was fairly common for sufferers of diabetes to experience like depression and low mood. And it was also reassuring to know that the experts who’d written the website or designed it or helped design it were aware of that as well, and you then think, well, I’m sure my doctor will, when I go and approach him about it that he’ll know too that it’s not just because of some random thing happening in my life that’s caused me to feel a little bit down or depressed, low mood, it’s also because I’m diabetic.

P6, female participant aged 46 years

However, some participants (2/19, 11%) reported having encountered very little of the emotional self-management content or did not recall this content at all (P5 and P7), primarily because they had stopped engaging with the structured education content early on during the program (P5).

### Theme 3: Experiences of Using Structured Education

Many participants (10/19, 53%) enjoyed working through a structured learning pathway and the ordering of the content in a logical progression (P1, P3, P7, P9, P10, P11, P13, P15, P16, and P17), which prevented them from becoming “sidetracked” (P17). Participants liked that information was presented to them in modules, so they could take in as much information as they needed at any one time (P2, P10, P14, and P15). This was particularly valued by participants who were newly diagnosed and acknowledged that it can feel like “information overload” (P9) at the start of their diagnosis and thus appreciated having sections of the website that they could work through systematically:

Well it was just it was in bite size chunks so I could pick a topic and finish it within ten or 15 minutes. I have lousy concentration, so it was good to be able to stop and not think, I’m going to lose my place now.

P10, female participant aged 43 years

It was never one huge meal to swallow, it was snacks. And you could have as many of those as you wanted at a time.

P14, male participant aged 69 years

However, the structured education did not suit everyone; some wanted the option to select topics of their choice (P5 and P18), and another participant disengaged once he encountered a section that was not applicable to him (P13):

But working through it, it started off getting started, what to know about diabetes, well, I’ve covered this, I want to be over there and I’m stuck here, and I think that may have been something that put me off going in further because it was like I haven’t got time for this, I need to know this. I need to click on subjects and then find what I want to know and listen to that rather than go from start to finish, because it got a bit boring, it did, and I think that’s why I stopped, because it got...it was too slow for me.

P5, female participant aged 56 years

After completing the structured education, most participants described wanting to use the website as an information tool as and when it was required, for example, to skim the contents to refresh memory on particular topics. However, others described feeling that after reading all the content on the website, it was no longer relevant (P11), or Healthy Living had been forgotten about over time (P5). Some participants (3/19, 16%) reported completing the structured education but not using it afterward (P11, P15, and P18) as follows:

In the longer term, the rest of this year where I’ve been bringing my weight down, the website didn’t seem to have any relevance. It sort of disappeared. When I couldn’t record stuff on it and I’d done all the training, it sort of...the relationship came to an end.

P11, male participant aged 75 years

### Theme 4: Importance of Technological, Professional, and Social Interactivity

#### Overview

In order to maximize the acceptability and continued engagement with Healthy Living in the longer term, participants suggested the need for more interactivity. This included increased technological interactivity between Healthy Living and other devices that they were already accessing (eg, wearable technology), interactivity from the website itself (eg, notifications), and interpersonal interactions both formally with health care professionals and informally with other people living with T2DM.

#### Subtheme 4.1: Interaction With Other Apps and Devices

Although Healthy Living included *Tools* for users to set goals and self-monitor their steps, weight, and hemoglobin A_1c_ level, participants reported that they did not use these Tools regularly. Instead, participants were already accustomed to using existing methods of self-monitoring via other apps and devices, which were not contingent with the Healthy Living website:

I did have a look at them [Tools]. And I think again for somebody who doesn’t have the access to other tools that I have, ideal. Absolutely ideal. But the Fitbit gives you the goals to set and it also wants your weight and your height and targets and everything.

P7, female participant aged 79 years

Therefore, participants already had a good understanding of techniques such as self-monitoring. They reported understanding the link between their behaviors (eg, diet and physical activity) and outcomes (eg, weight and blood glucose levels), helping them adequately self-regulate their health behaviors as part of their T2DM medical self-management (P3, P14, and P16).

These digital tools that the participants were already using outside of Healthy Living logged their behaviors automatically and provided feedback, which was a valuable part of their self-management (P1, P4, P5, P6, P12, and P17). Consequently, some participants (3/19, 16%) wanted Healthy Living to provide more personalized feedback, similar to what their existing tools and resources were already offering (P1 and P11) but with more tailored recommendations in relation to their T2DM self-management (P1 and P5) to encourage more interactivity with the website:

...[I]f there was some sort of feedback perhaps from the Healthy Living site that just says, well [Name], we’ve not progressed very well, there are these 12 different things that you might want to try and improve on. But there isn’t that sort of feedback at the moment which I think would be helpful, I really do.

P1, male participant aged 74 years

Therefore, it was suggested that Healthy Living could be more useful as an app (P5, P10, P12, and P17) to enable better integration of the education provided by Healthy Living with the existing apps that participants were already using on their phones. Participants felt that complementarity with other technologies could prevent users from forgetting about the website over time:

So again it needs to be connected to something that’s in my face, that works like that...as I say I use Samsung Health, I do my exercise with it. When I walk I switch it on then I know that does me good but then if that would automatically log with that I wouldn’t have to go in, oh, well, I’ve walked this much today. Because you forget, you’ve walked, you don’t think I’ll get home and I’m going to go and log that, but because the app does it automatically, just say walking it follows me and it does it, and that’s what it needs to do. It would be perfect if it did that.

P5, female participant aged 56 years

It was also suggested that more interactivity from the Healthy Living website itself would help improve the experience and continued use. For example, some participants suggested email nudges (P7 and P11) and notifications (P13) from Healthy Living to keep people engaged with the program over time.

#### Subtheme 4.2: Interaction With Health Care Professionals

It was noted that the lack of health care professional support from Healthy Living meant that nobody was monitoring the website to review service users’ progress with the program:

And I have a problem sometimes getting self-motivated...And that’s what I’m conscious of, in terms of the website...There’s actually no one there that I’ve got to see every week to review what I’ve done. I’ve got to do it myself.

P19, male participant aged 70 years

While some participants (6/19, 32%) used Healthy Living to support conversations with their health care professionals outside of the program (P1, P4, P6, P13, P14, and P16), including discussions around their emotional self-management, it was also acknowledged that other people living with T2DM may not receive the same level of support from their local health service and thus may rely more on the support from Healthy Living:

So yeah, maybe...I’ve got a particularly good diabetic nurse...But yeah, I count myself lucky in that sense. And so maybe some of the things that other people might need from a programme like this, I’m already getting elsewhere.

P14, male participant aged 69 years

Participants provided suggestions on how to improve the interactivity with health care professionals via the Healthy Living website. These included live webinars (P5), a question and answers section (P6), and a Healthy Living email address to submit questions to health care professionals (P13). Other participants suggested the functionality to link Healthy Living to their GP practice systems to enable GP practices to access the data inputted into Healthy Living and to guide conversations with health care professionals at upcoming appointments (P5, P10, and P16). Most participants (14/19, 74%) did not feel that they needed any form of facilitated access from a health care professional when first signing up to Healthy Living, as they felt the website was easy to understand without this additional support.

#### Subtheme 4.3: Interaction With Other People Living With T2DM

In response to a question about whether there was a need for an online support forum in Healthy Living, some participants (4/19, 21%) said they would have liked the opportunity to interact with other people living with T2DM (P7, P8, P9, and P12):

You know, like, if you’re on Diabetes UK you’ve got forums and things and there isn’t...that would be a useful addition I think to this, would be to have some, kind of, forum where people can network a little bit.

P9, female participant aged 54 years

However, there was a concern that a forum might spread misinformation, and participants compared this to websites such as Facebook, and to avoid this, it would have to be moderated by health care professionals (P5, P10, P11, P13, P16, P17, and P18):

I suppose that [group forum] would be good but again it’s got to be managed to make sure it’s reliable information. My go to website is, if I don’t find what I want on the NHS website is Diabetes UK. I don’t go to Facebook pages anymore I learnt that lesson years ago because you just get chatter and you get, don’t do that, I do this and ends up with arguments and false information or drug names getting confused and misspelt. So there’s definitely a need for more reliable information for patients, especially as the web grows and more and more people are using smartphones.

P10, female participant aged 43 years

I probably wouldn’t bother [with an online forum] because I find places like Facebook and Twitter, people, a large group of people say things that are actually wrong.

P18, male participant aged 65 years

The videos embedded into the structured education content about other people’s stories living with T2DM offered an opportunity for peer support for some participants (P2, P3, P4, P6, P11, P15, and P17). This gave them the opportunity to “listen to other people’s experiences” (P11) and “sympathize” with others on the “same journey” as them (P3), which in turn validated their own experiences of living with T2DM. Some newly diagnosed participants reported these videos to be especially useful (P2, P3, and P17). Therefore, although not a live interaction with others, the videos provided a form of support that some participants benefited from:

...[I]t’s almost like having your chat group, but with people with videos, because I actually learn well via videos as opposed to reading, and I just thought people have got similar problems, and they’re all talking about it and how they cured it and their problems. I just found that was really very good empathy for me.

P17, female participant aged 61 years

## Discussion

### Principal Findings

Service users valued Healthy Living, as it provided them with a reliable source of information, which they could access when they needed to as part of their T2DM self-management. The emotional self-management content particularly resonated with some participants, prompting them to book an appointment with their GP or nurse to discuss their low mood. Participants suggested that they might have been encouraged to use the website in the longer term if there was more interactivity with the website. These aspects of interactivity included (1) interaction with the existing technologies and the website itself, (2) formal interaction with health care professionals and services for T2DM self-management, and (3) informal interactivity with other people for social support. Although most participants reported finding some information on Healthy Living useful, there was consensus that the website was particularly suitable for those newly diagnosed with T2DM.

### Strengths and Limitations

This study presented a unique opportunity to assess service user experiences of a digital DSMES program that has demonstrated effectiveness in a trial and is being rolled out nationally across England. Efforts were made to secure a broad representation of participants across age, sex, ethnic groups, and length of diagnosis, although the sample had little ethnic diversity, which was reflective of the sample of people using Healthy Living at the time of the interviews. The median age of participants in this study was 61 (IQR 53-73, range 43-81) years; however, younger participants may have had different perceptions of the program. Given that the recruitment for this study took place during the COVID-19–related restrictions and that the participants who had used the program would have used it during the pandemic, these may have impacted people’s engagement with the program and subsequent recruitment to the study.

We deliberately spoke with the most engaged users, with the intention of interviewing those who had used a sufficient amount of the website content to allow an in-depth understanding of how people were using the digital program. The current sample of participants was useful for the purpose of this study; however, other samples of service users (eg, those who are less engaged or who did not take up the program) would have different views on some aspects of the intervention. Therefore, the current results are more applicable to people who are more engaged with (1) their own T2DM self-management and (2) using digital interventions. However, in cases where this sample of engaged participants reported not using components of Healthy Living, it provides a strong argument for where improvements could be made to the program to increase engagement.

### Comparison With Prior Work

There were 3 key changes in the implementation of Healthy Living into routine care since the HeLP-Diabetes RCT [18]. First, due to changes in the NHS policy, Healthy Living included a structured education component that service users had to work through in a linear fashion. Prior research found that users with long-term health conditions preferred to have control over what topics they accessed for information at any one time, and those who were already knowledgeable about their condition preferred to be provided with in-depth information [43]. Improvements have since been made to Healthy Living so that it is clearer for service users that they can either complete the structured element or choose their own topics via the unstructured education element of the program. Second, Healthy Living did not incorporate facilitated access into the program (ie, where a health care professional helps users to sign up and access the program) due to (1) challenges in scaling up the HeLP-Diabetes RCT into routine practice and (2) updated access to Healthy Living since the RCT, which had improved usability for low digital literacy. Therefore, the program is entirely self-led [18]. Previous qualitative research has reported that participants were strongly in favor of health care professionals providing support for how to use the website in the HeLP-Diabetes RCT [44], although participants in this study felt the website was self-explanatory and easy to use.

Third, Healthy Living did not include an online peer-support forum due to the low uptake of this feature in the original RCT, so there was insufficient evidence that justified the cost of delivering it at scale [18]. Previous qualitative work exploring service users’ experiences of using the HeLP-Diabetes RCT reported that some felt “part of a community” with the inclusion of an online forum and valued the opportunity to interact with others on the website [37]. Participants in this study felt that an online forum would only be a useful addition to the website if it was moderated by health care professionals to prevent the spread of misinformation. Despite this perspective, there is much evidence in the literature highlighting the importance of online forums for people with T2DM; for example, service users have reported drawing on shared experiences from others, which empowered them to engage with health care services [45]. Given the underpinning evidence, intervention developers of digital DSMES programs could consider signposting service users to other group forums (eg, Diabetes UK) if they lack the resource to run their own moderated peer-support forum.

Participants in this study found the emotional management content valuable. It is particularly noteworthy that some participants who were already engaged with their T2DM self-management were still unaware of the link between their T2DM and low mood, and this prompted them to book an appointment to discuss with a health care professional, which was an intended purpose of the website [18]. In this context, participants were not receiving emotional support via interaction but valued the informational support that they received about the emotional impact of illness and how to manage it. Previous qualitative research found that emotional support was valued for T2DM self-management [16] and self-management training for other chronic illnesses [46]. Research has also highlighted that people living with T2DM find it difficult to manage their emotions and adapt to changes in their lifestyle after receiving their diagnosis [47]. Given the calls to prioritize the psychological well-being of people living with T2DM [48], there is the argument to include emotional management content earlier on in the T2DM self-management program curricula to reduce the risk of users missing this important content if they disengage from the structured education. Since this study was conducted, NHS England has made improvements to signposting to emotional well-being content in the nonstructured part of the program to allow service users to access some content without needing to work through the structured education element of Healthy Living.

Participants suggested that Healthy Living would be more useful as an app that is immediately accessible on their phones to increase the ease of access and enable interaction with other technologies that they were regularly using as part of their self-management; similar findings have been reported previously [16]. Thus, interaction with the existing technologies seems important in order for an informational website to complement what people are already doing to self-manage their T2DM. Further interactivity from the website itself, including more tailored feedback on a person’s T2DM self-management, could also promote continued engagement. User engagement research has found that sending a push notification containing a tailored health SMS text message was associated with greater engagement in a mobile health app [49], and apps that were tailored to users’ preferences and contained personalized feedback resulted in continued engagement [50,51]. Thus, to sustain engagement with digital DSMES programs in the longer term, intervention developers could consider ways to increase the interconnectivity both within the interventions (eg, via notifications and prompts) and with existing technologies. NHS England has since implemented notifications on the Healthy Living website following this interview study.

### Implications

There was consensus across participants that they would recommend Healthy Living to those who are newly diagnosed, and many felt that the website was especially useful for this group of people. Interviewees newly diagnosed with T2DM also expressed that they would have liked access to this website as soon as they were informed about their diagnosis. Furthermore, participants reported to learn something new from the website, even if they had used face-to-face services in the past. Thus, there is a need for a clear pathway in primary care to establish where Healthy Living fits with the other DSMES programs. For example, general practices could be encouraged to inform people about Healthy Living as soon as they receive their T2DM diagnosis, which could work in conjunction with the face-to-face DSMES programs on offer. The face-to-face sessions could offer the opportunity for (1) formal interaction with other professionals and services for managing T2DM and (2) social and informal support from peers, while the website could allow service users to obtain informational support and work through the educational content at their own pace. Further research could also explore which content is most useful for those who have been living with T2DM for a longer period of time to promote self-management maintenance.

This study explicitly aimed to obtain the views of service users who had sufficient engagement with Healthy Living. Thus, future research may need to use other sampling processes to assess how the intervention could be modified to limit digital exclusion, avoid exacerbating health inequalities, and assess whether Healthy Living meets the needs of people from different ethnic groups. Another fruitful avenue for further research would be to interview service users who either chose not to take up the Healthy Living program or stopped using the program early on. Future research could also speak to people at the point of referral in primary care about their experiences of being referred to a program like Healthy Living, exploring reasons why people may choose to take up a self-management program and what support is required at referral [52]. Such research could also help to understand any potential inequalities with access to digital interventions, such as Healthy Living, and whether inequalities might be increased.

The participants in this study were more engaged in the use of Healthy Living, so they may also be more likely to have engaged with other tools and technologies outside of the program. For users who do not have access to other tracking tools, Healthy Living may be more useful. It would therefore be informative to interview people who do not otherwise have access to external tracking tools and devices, to establish whether the self-regulatory Tools on Healthy Living are providing value for this group of people. The assessment of usage data would provide an understanding of the use of these tools for all users enrolled in Healthy Living and shed light on the extent to which the users are engaging with the structured education content and where a drop-off in engagement might occur.

### Conclusions

This study offers valuable insights into service users’ experiences of a nationally implemented digital DSMES program. Digital DSMES programs offering emotional aspects of self-management are addressing an unmet need. Healthy Living was of most value as a trusted source of information, in particular, to those who were newly diagnosed with T2DM. Primary care could usefully offer digital DSMES programs to people as soon as they are diagnosed.

## Appendix

Multimedia Appendix 1 Healthy Living website content.

Multimedia Appendix 2 Standards for Reporting Qualitative Research.

Multimedia Appendix 3 REDCap (Research Electronic Data Capture) questionnaire.

Multimedia Appendix 4 Criteria for participant recruitment.

Multimedia Appendix 5 Interview topic guide.

Multimedia Appendix 6 Coding framework.

Multimedia Appendix 7 Thematic structure.

## Abbreviations

DSMES: Diabetes Self-Management Education and Support
GP: general practitioner
HeLP-Diabetes: Healthy Living for People with Type 2 Diabetes
NHS: National Health Service
PPIE: Patient and Public Involvement and Engagement
RCT: randomized controlled trial
REDCap: Research Electronic Data Capture
T2DM: type 2 diabetes mellitus

## Data Availability

Some data sets are available from the corresponding author on reasonable request, although authors will require the explicit permission of the relevant external organizations.

## References

[1] (2015). Type 2 diabetes in adults: management guideline. National Institute for Health and Care Excellence.

[2] Chatterjee S, Davies MJ, Heller S, Speight J, Snoek FJ, Khunti K (2018). Diabetes structured self-management education programmes: a narrative review and current innovations. Lancet Diabetes Endocrinol.

[3] Norris SL, Engelgau MM, Narayan KM (2001). Effectiveness of self-management training in type 2 diabetes: a systematic review of randomized controlled trials. Diabetes Care.

[4] Panagioti M, Richardson G, Small N, Murray E, Rogers A, Kennedy A, Newman S, Bower P (2014). Self-management support interventions to reduce health care utilisation without compromising outcomes: a systematic review and meta-analysis. BMC Health Serv Res.

[5] Davies MJ, Heller S, Skinner TC, Campbell MJ, Carey ME, Cradock S, Dallosso HM, Daly H, Doherty Y, Eaton S, Fox C, Oliver L, Rantell K, Rayman G, Khunti K (2008). Effectiveness of the diabetes education and self management for ongoing and newly diagnosed (DESMOND) programme for people with newly diagnosed type 2 diabetes: cluster randomised controlled trial. BMJ.

[6] Deakin TA, Cade JE, Williams R, Greenwood DC (2006). Structured patient education: the diabetes X-PERT programme makes a difference. Diabet Med.

[7] Li R, Shrestha SS, Lipman R, Burrows NR, Kolb LE, Rutledge S, Centers CDC (2014). Diabetes self-management education and training among privately insured persons with newly diagnosed diabetes--United States, 2011-2012. MMWR Morb Mortal Wkly Rep.

[8] (2018). National diabetes audit 2016/2017. National Health Service Digital.

[9] Howarth E, Bower PJ, Kontopantelis E, Soiland-Reyes C, Meacock R, Whittaker W, Cotterill S (2020). 'Going the distance': an independent cohort study of engagement and dropout among the first 100 000 referrals into a large-scale diabetes prevention program. BMJ Open Diabetes Res Care.

[10] Reeves D, Woodham AA, French DP, Bower P, Holland F, Kontopantelis E, Cotterill S (2023). The influence of demographic, health and psychosocial factors on patient uptake of the English NHS diabetes prevention programme. BMC Health Serv Res.

[11] Horigan G, Davies M, Findlay-White F, Chaney D, Coates V (2017). Reasons why patients referred to diabetes education programmes choose not to attend: a systematic review. Diabet Med.

[12] Ross J, Cotterill S, Bower P, Murray E (2023). Influences on patient uptake of and engagement with the national health service digital diabetes prevention programme: qualitative interview study. J Med Internet Res.

[13] (2019). The NHS long term plan. National Health Service, England.

[14] Murray E, Sweeting M, Dack C, Pal K, Modrow K, Hudda M, Li J, Ross J, Alkhaldi G, Barnard M, Farmer A, Michie S, Yardley L, May C, Parrott S, Stevenson F, Knox M, Patterson D (2017). Web-based self-management support for people with type 2 diabetes (HeLP-Diabetes): randomised controlled trial in English primary care. BMJ Open.

[15] Dack C, Ross J, Stevenson F, Pal K, Gubert E, Michie S, Yardley L, Barnard M, May C, Farmer A, Wood B, Murray E (2019). A digital self-management intervention for adults with type 2 diabetes: combining theory, data and participatory design to develop HeLP-Diabetes. Internet Interv.

[16] Poduval S, Marston L, Hamilton F, Stevenson F, Murray E (2020). Feasibility, acceptability, and impact of a web-based structured education program for type 2 diabetes: real-world study. JMIR Diabetes.

[17] (2019). Healthy living service specification. National Health Service, England.

[18] Benton JS, Cotterill S, Hawkes RE, Miles LM, French DP (2022). Changes in a digital type 2 diabetes self-management intervention during national rollout: mixed methods study of fidelity. J Med Internet Res.

[19] Murray E, Ross J, Pal K, Li J, Dack C, Stevenson F, Sweeting M, Parrott SJ, Barnard M, Yardley L, Michie S (2018). A web-based self-management programme for people with type 2 diabetes: the HeLP-diabetes research programme including RCT. Programme Grants Appl Res.

[20] Michie S, Richardson M, Johnston M, Abraham C, Francis J, Hardeman W, Eccles MP, Cane J, Wood CE (2013). The behavior change technique taxonomy (v1) of 93 hierarchically clustered techniques: building an international consensus for the reporting of behavior change interventions. Ann Behav Med.

[21] Corbin JM, Strauss A (1988). Unending Work and Care: Managing Chronic Illness at Home.

[22] (2014). Five year forward view. National Health Service, England.

[23] Linke S, McCambridge J, Khadjesari Z, Wallace P, Murray E (2008). Development of a psychologically enhanced interactive online intervention for hazardous drinking. Alcohol Alcohol.

[24] Wallace P, Murray E, McCambridge J, Khadjesari Z, White IR, Thompson SG, Kalaitzaki E, Godfrey C, Linke S (2011). On-line randomized controlled trial of an internet based psychologically enhanced intervention for people with hazardous alcohol consumption. PLoS One.

[25] Yardley L, Williams S, Bradbury K, Garip G, Renouf S, Ware L, Dorling H, Smith E, Little P (2012). Integrating user perspectives into the development of a web-based weight management intervention. Clin Obes.

[26] Yardley L, Ware LJ, Smith ER, Williams S, Bradbury KJ, Arden-Close EJ, Mullee MA, Moore MV, Peacock JL, Lean ME, Margetts BM, Byrne CD, Hobbs RF, Little P (2014). Randomised controlled feasibility trial of a web-based weight management intervention with nurse support for obese patients in primary care. Int J Behav Nutr Phys Act.

[27] Michie S, Hyder N, Walia A, West R (2011). Development of a taxonomy of behaviour change techniques used in individual behavioural support for smoking cessation. Addict Behav.

[28] Brown J, Michie S, Geraghty AW, Yardley L, Gardner B, Shahab L, Stapleton JA, West R (2014). Internet-based intervention for smoking cessation (StopAdvisor) in people with low and high socioeconomic status: a randomised controlled trial. Lancet Respir Med.

[29] Pittaway S, Cupitt C, Palmer D, Arowobusoye N, Milne R, Holttum S, Pezet R, Patrick H (2009). Comparative, clinical feasibility study of three tools for delivery of cognitive behavioural therapy for mild to moderate depression and anxiety provided on a self-help basis. Ment Health Fam Med.

[30] Herxheimer A, McPherson A, Miller R, Chapple A, Shepperd S, Ziebland S, Sanz E (2003). DIPEx (database of individual patients experience of illness): a multimedia proposal to share experiences and information about illnesses between patients and health professionals [Article in Spanish]. Aten Primaria.

[31] (2019). Government functional standard GovS 005: digital. UK Government.

[32] Web content accessibility guidelines (WCAG) 2.1. World Wide Web Consortium (W3C).

[33] (2021). The digital technology assessment criteria for health and social care (DTAC). National Health Service, England.

[34] Content style guide. National Health Service, England.

[35] NHS Digital style guidelines. National Health Service, England.

[36] Hoffmann TC, Glasziou PP, Boutron I, Milne R, Perera R, Moher D, Altman DG, Barbour V, Macdonald H, Johnston M, Lamb SE, Dixon-Woods M, McCulloch P, Wyatt JC, Chan A, Michie S (2014). Better reporting of interventions: template for intervention description and replication (TIDieR) checklist and guide. BMJ.

[37] Hofmann M, Dack C, Barker C, Murray E (2016). The impact of an internet-based self-management intervention (HeLP-Diabetes) on the psychological well-being of adults with type 2 diabetes: a mixed-method cohort study. J Diabetes Res.

[38] Bellg A, Borrelli B, Resnick B, Hecht J, Minicucci DS, Ory M, Ogedegbe G, Orwig D, Ernst D, Czajkowski S (2004). Enhancing treatment fidelity in health behavior change studies: best practices and recommendations from the NIH Behavior Change Consortium. Health Psychol.

[39] O'Brien BC, Harris IB, Beckman TJ, Reed DA, Cook DA (2014). Standards for reporting qualitative research: a synthesis of recommendations. Acad Med.

[40] Home page. RedCap.

[41] Gale NK, Heath G, Cameron E, Rashid S, Redwood S (2013). Using the framework method for the analysis of qualitative data in multi-disciplinary health research. BMC Med Res Methodol.

[42] (2019). The English indices of deprivation. Department for Communities and Local Government.

[43] Kerr C, Murray E, Stevenson F, Gore C, Nazareth I (2006). Internet interventions for long-term conditions: patient and caregiver quality criteria. J Med Internet Res.

[44] Pal K, Dack C, Ross J, Michie S, May C, Stevenson F, Farmer A, Yardley L, Barnard M, Murray E (2018). Digital health interventions for adults with type 2 diabetes: qualitative study of patient perspectives on diabetes self-management education and support. J Med Internet Res.

[45] Brady E, Segar J, Sanders C (2017). Accessing support and empowerment online: the experiences of individuals with diabetes. Health Expect.

[46] Sanders C, Rogers A, Gardner C, Kennedy A (2011). Managing 'difficult emotions' and family life: exploring insights and social support within online self-management training. Chronic Illn.

[47] Berenguera A, Molló-Inesta À, Mata-Cases M, Franch-Nadal J, Bolíbar B, Rubinat E, Mauricio D (2016). Understanding the physical, social, and emotional experiences of people with uncontrolled type 2 diabetes: a qualitative study. Patient Prefer Adherence.

[48] Jones A, Vallis M, Pouwer F (2015). If it does not significantly change HbA1c levels why should we waste time on it? a plea for the prioritization of psychological well-being in people with diabetes. Diabet Med.

[49] Bidargaddi N, Almirall D, Murphy S, Nahum-Shani I, Kovalcik M, Pituch T, Maaieh H, Strecher V (2018). To prompt or not to prompt? a microrandomized trial of time-varying push notifications to increase proximal engagement with a mobile health app. JMIR Mhealth Uhealth.

[50] Anderson K, Burford O, Emmerton L (2016). Mobile health apps to facilitate self-care: a qualitative study of user experiences. PLoS One.

[51] Bults M, van Leersum CM, Olthuis TJ, Bekhuis RE, den Ouden ME (2023). Mobile health apps for the control and self-management of type 2 diabetes mellitus: qualitative study on users' acceptability and acceptance. JMIR Diabetes.

[52] Howells K, Bower P, Burch P, Cotterill S, Sanders C (2021). On the borderline of diabetes: understanding how individuals resist and reframe diabetes risk. Health Risk Soc.

