# Supplementary material for: Service Users’ Experiences of a Nationwide Digital Type 2 Diabetes Self-Management Intervention (Healthy Living): Qualitative Interview Study
Source: JMIR Diabetes. 2024 Jul 18;9:e56276. doi: 10.2196/56276 (PMC11294771; doi:10.2196/56276)
Supplement: Multimedia Appendix 1 [file diabetes_v9i1e56276_app1.docx]

**Multimedia Appendix 1****: Healthy Living website content**

Screenshots of the three main components in the Healthy Living website: ‘Learn’ (top), ‘Find answers’ (middle) and ‘Tools’ (bottom).


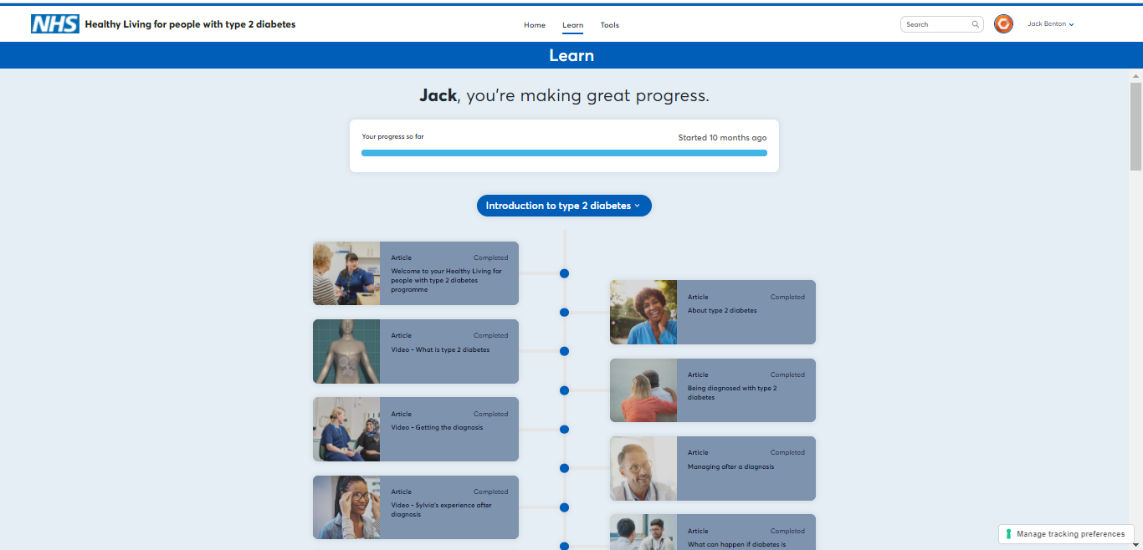


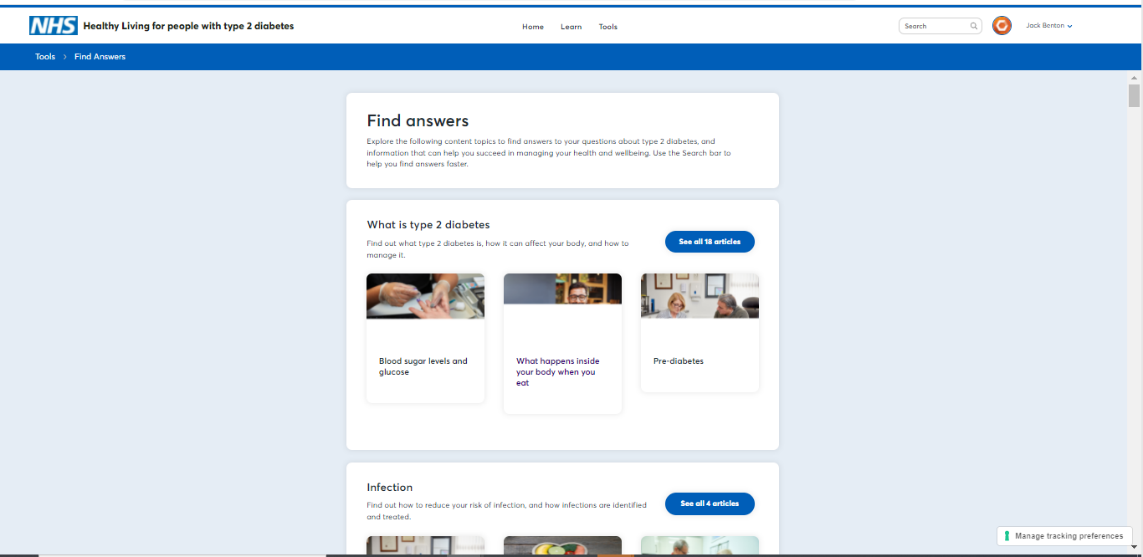


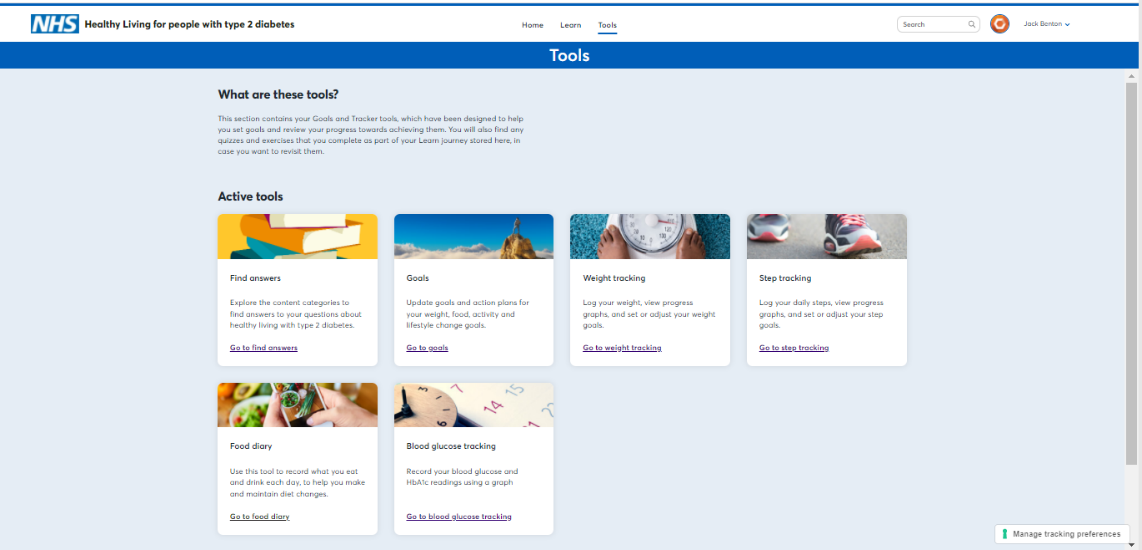


**Description of Healthy Living using the TIDieR framework *^a^***

| **TIDieR item *^b^*** | **Healthy Living for people with type 2 diabetes Programme (Healthy Living)** |
| --- | --- |
| Why (Rationale) | The principal strategic drivers for this project were the NHS England Five Year Forward View [22] and to deliver the following Structured Education and Treatment target indicators:   - Diabetes patients who have achieved all of the NICE-recommended treatment targets (Three targets for adults-HbA1c, cholesterol and blood pressure: one target for children-HbA1c); - People with diabetes diagnosed less than a year ago who attend a structured education course.   The investment seeks to deliver on NHS England’s strategy to harness information and technology to support individuals to manage their care by developing a low-cost service to support individuals with T2DM. |
| What (Materials) | Healthy Living is a free digital NHS service for people living with T2DM to support them to self-manage their condition. The service can be used on a range of digital devices (i.e. smartphones, desktops, tablets). The website contains 895 web pages.  Website content is broken down into three main sections (see screenshots above):   1. Learn (structured curriculum based on the HeLP-Diabetes: Starting Out structured course [16]) – 273 pages split into 26 modules; 2. Find answers (sections dedicated to different topics relating to T2DM where users could dip in and out of different pages and sections, based on the HeLP-Diabetes website [14]) – 583 pages split into 37 sections; 3. Tools (interactive tools) – 39 pages including goal setting, action planning and self-monitoring tools.   Content includes information about what type 2 diabetes is, its causes and how it can be managed and treated; behavioural advice on diet, physical activity, alcohol, smoking and medication adherence; and emotional and practical support. There are interactive tools for users to set and review goals and action plans for weight, diet, physical activity, daily steps, alcohol, medicines and wellbeing. There are also self-monitoring tools for weight, daily steps, diet and blood glucose.  A content analysis of the Healthy Living website [18] identified 43 behaviour change techniques and all areas of self-management (medical, emotional, and role) were addressed throughout the webpages.  Pre-existing interventions that are incorporated into Healthy Living included DownYourDrink [23, 24], POWeR [25, 26], StopAdvisor [27, 28], Living Life to the Full [29], and HealthTalkOnline personal video stories [30]. Users are also sent communication to promote engagement via emails and notifications. No physical materials are offered to users. |
| What (Procedures) | Healthy Living is intended for people diagnosed with T2DM in England, carers and healthcare professionals, available by self-referral and signposting from primary care. Users register online by visiting the self-registration page of the website. Users can read articles; watch videos; complete self-assessment quizzes; set, plan and review goals and identify potential barriers to achieving these goals and ideas for navigating these barriers. Users can opt-in to receive notifications. Users can submit an online form for technical support or ring the support helpline number, but there are no health care professionals involved in supporting registration or use of the website (i.e. facilitated access). |
| Who provided | An external digital service provider was commissioned to develop and provide this NHS service. NHS England assigned a project manager from the NHS England Diabetes Team to manage programme delivery according to the procurement contract. Healthy Living has NHS branding and gave the impression to users of being an NHS service. Technical support is provided by an administrator from the digital service provider to help people with technical problems such as lost passwords and to coordinate additional support from relevant teams within the service provider as required. Coaching or individual clinical advice is not offered in Healthy Living. |
| How (modes of delivery) | The service is delivered online for individuals. |
| Where | The service can be used wherever people have a digital device and internet access. |
| When and how much | Once registered, service users can use the website as much (or as little) over any period of time that suits them. However, service users are encouraged to use the structured ‘Learn’ curriculum. Modules in this curriculum can be completed in one sitting, or progress can be saved and users could resume at any time. NHS England specified that service users who progress through the first 60% of the ‘Learn’ curriculum are classified as having completed the programme. This threshold was agreed upon by the policy team based on benchmarking completion against other similar services and structured education programmes. |
| Tailoring | There is a small amount of tailoring based on questions after registering, which filters content relevant to the user’s smoking, employment and driving status. Written feedback is provided to users who complete the self-assessment quizzes, using validated tool calculations to provide responses based on their answers. These responses would indicate to the user any recommended actions the user might need to take to address their resulting score. This could include signposting to sections of the content or external services, suggestions of actions to take such as increasing physical activity or reducing alcohol consumption, and prompting setting goals in the programme to help achieve these recommended changes. |
| Modifications | The website is being developed on an ongoing basis to improve usability, accessibility and user engagement with the service. Modifications are completed in line with user research and feedback, and guidelines including the Government Digital Service (GDS) Standard [31], Web Content Accessibility Guidelines (WCAG) [32], Digital Technology Assessment Criteria [33] and NHS Digital Content [34] and Style Guidelines [35]. |

^a^ Table adapted from Benton et al. [18].
^b^ Items correspond to the headings from the Template for Intervention and Description Replication (TIDieR) framework [36].
