# Supplementary material for: Service Users’ Experiences of a Nationwide Digital Type 2 Diabetes Self-Management Intervention (Healthy Living): Qualitative Interview Study
Source: JMIR Diabetes. 2024 Jul 18;9:e56276. doi: 10.2196/56276 (PMC11294771; doi:10.2196/56276)
Supplement: Multimedia Appendix 3 [file diabetes_v9i1e56276_app3.pdf]

You are being invited to complete this online survey to register your interest to take part in a paid research interview about your experience of the ‘Healthy Living’ diabetes programme.

Before you decide whether or not to register your interest to take part, please take time to read the following information about this research:Study Information Sheet

If you would like to be considered for an interview, please complete the survey questions below.

We are asking these questions because we want to make sure that we hear from a wide range of people with type 2 diabetes.

If you are chosen to take part in an interview you will be paid for your time.

(Please note that completing this survey does not guarantee that you will be selected for an interview)

What is your gender?

◯ Male
◯ Female
◯ Other

What is your age group?

◯ 18 to 29
◯ 30 to 39
◯ 40 to 49
◯ 50 to 59
◯ 60 to 69
◯ 70 or above

Which one of the following best describes your ethnic group or background?

◯ White
◯ Mixed ethnic background (e.g. White and Asian, White and Black African)
◯ Asian or Asian British (includes Indian, Pakistani or Bangladeshi)
◯ Black or Black British (includes Caribbean or African)
◯ Chinese
◯ Other ethnic group (please specify)

Please tell us your ethnic group

____

What is your postcode? (This is so that we can select a range of people who live in wealthy and deprived areas)

____

How long ago were you diagnosed with type 2 diabetes by a health professional?

◯ Less than a year
◯ Between 1 and 2 years
◯ More than 2 years

Contact details (We will only use this information to contact you if you are selected for an interview)

Name

____

Please provide your email address or telephone number

____

If you are selected to take part in an interview, you will be contacted by a researcher from the University of Manchester by email or telephone within 14 days.
