# Supplementary material for: Service Users’ Experiences of a Nationwide Digital Type 2 Diabetes Self-Management Intervention (Healthy Living): Qualitative Interview Study
Source: JMIR Diabetes. 2024 Jul 18;9:e56276. doi: 10.2196/56276 (PMC11294771; doi:10.2196/56276)
Supplement: Multimedia Appendix 4 [file diabetes_v9i1e56276_app4.docx]

**Multimedia Appendix 4 – Details of criteria for participant recruitment**

The service provider was able to use email triggers based on either a specific user action in the programme (e.g. viewing content, accessing a plugin, logging data, completing a goal), or number of days since the user first accessed the programme. This was to ensure they were contacting service users who had recently engaged with the Healthy Living programme to a sufficient degree and to ensure that the service users being interviewed had used the most up to date version of the programme. Emails could also be filtered by user characteristics (e.g. ethnicity), to enable researchers to target particular subgroups. Example email triggers included: completion of at least 60% of the Learn journey (structured component) to date; completion of a goal since February 2021; non-White ethnicity. As there was a limited pool of participants to sample from, the email triggers changed throughout the recruitment process to aim to achieve diversity in the final sample of interviewees.

We started off by targeting people who had completed at least 60% of the Learn Journey; this threshold was determined by NHS England as ‘completion’ of the programme. However, we relaxed this criteria to 30% during recruitment as the numbers of people who had completed at least 60% of the programme were low at the time this study was conducted. The criteria of 30% was decided in collaboration with NHS England and the service provider.

See flow diagram below.

**Total: n=19 participants**

4 participants recruited

2 participants recruited

5 participants recruited

1 participant recruited

3 participants recruited

4 participants recruited

Target all users who have been emailed about the study to date with a reminder

432 emails sent on 3^rd^ December 2021

Target all users who have completed 30% of structured Learn journey since January 2021

212 emails sent on 26^th^ November 2021

Target all users who have set a goal since February 2021

221 emails sent on 19^th^ November 2021

Target users diagnosed with type 2 diabetes < 12 months AND [have completed at least 30% of structured Learn journey OR set a goal]

83 emails sent on 12^th^ November 2021

Target NON-WHITE users only, participants who had completed at least 30% of the structured Learn journey OR set a goal

17 emails sent on 5^th^ November 2021

Target participants who had completed 60% of the structured Learn journey

108 emails sent on 15^th^ and 29^th^ October 2021
