# Supplementary material for: Service Users’ Experiences of a Nationwide Digital Type 2 Diabetes Self-Management Intervention (Healthy Living): Qualitative Interview Study
Source: JMIR Diabetes. 2024 Jul 18;9:e56276. doi: 10.2196/56276 (PMC11294771; doi:10.2196/56276)
Supplement: Multimedia Appendix 5 [file diabetes_v9i1e56276_app5.docx]

**Multimedia Appendix 5 – Interview Topic Guide**

1. To begin with then, can you tell me how you found out about the online ‘Healthy Living’ diabetes programme?

- When did you first register for the Healthy Living programme?

1. How often have you been using this online programme so far? (e.g. every day, every week, as and when)
   - What device do you access the programme from? E.g. a desktop computer, laptop, tablet, mobile phone
2. How easy have you found it to use the online programme?

- What was easy?
- What was difficult?
- Was it clear how you should use the programme?
- Was there anything specific you wanted to get out of using the programme?
- Is it similar or different to what you were expecting?

1. Which sections of the online programme have you found most useful?

**[prompt sections: Learn section, Find Answers, Interactive tools, Topics (Medical/Emotional/Role)]**

- What do you like about this section?
- What did you not like about this section?
- Did you like the way the Learn journey was structured?
- What do you think this section was trying to achieve?
- Do you think it achieved this for you?
- Why did you continue to use this section (what kept them engaged)?
- How has your use of the programme changed over time? (especially since completing the Learn section)
  - What did you use more/ less over time?
  - Why?

1. Have you used any of the **‘Goals’ tools** (in the ‘Tools’ section of the programme)?

- Weight goal
- Diet goal
- Physical activity goal
- Alcohol goal
- Medicine goal
- Wellbeing plan
- Daily steps
- Red Food goal

(If yes, ask 6 to 9)

1. What goals have you set using the **‘Goals’ tool**? *[explain if necessary]*
   - How are you getting on with that? (Choosing, setting, working towards goal?)
   - How do you think setting goals might help people to change their [weight, diet, physical activity, alcohol intake, taking medicine, wellbeing, daily steps]?
   - How useful are you finding setting goals for changing your [weight, diet, physical activity, alcohol intake, taking medicine, wellbeing, daily steps]?
2. Have you used the programme to make a **detailed plan** for how you would achieve your goals? *[explain if necessary]*
   - How are you getting on with that? (Making a plan, sticking to it?)
   - How do you think these plans might help people to change their [weight, diet, physical activity, alcohol intake, taking medicine, wellbeing, daily steps]?
   - How useful are you finding making those plans for changing your [weight, diet, physical activity, alcohol intake, taking medicine, wellbeing, daily steps]?
3. Have you used the programme to think about any **barriers** that get in the way of achieving your goals and come up with solutions to get over those barriers? *[explain if necessary]*
   - How did you get on with that?
   - How do you think overcoming barriers in this way might help people to change their [weight, diet, physical activity, alcohol intake, taking medicine, wellbeing, daily steps]?
   - How useful did you find this for changing your [weight, diet, physical activity, alcohol intake, taking medicine, wellbeing, daily steps]?
4. Have you used the programme to **review your goals**? *[explain if necessary]*
   - How did you get on with that?
   - How do you think reviewing your goals in this way might help people to change their [weight, diet, physical activity, alcohol intake, taking medicine, wellbeing, daily steps]?
   - How useful did you find this for changing your [weight, diet, physical activity, alcohol intake, taking medicine, wellbeing, daily steps]?
5. Have you used any of the **‘Tracking’ tools** (in the ‘Tools’ section of the programme)?

- Weight tracking
- Step tracking
- Food diary
- Blood glucose tracking

(If yes, ask 11 and 12)

1. What **tracking tools** have you been using in the programme?
   - How are you getting on with that?
   - How do you think keeping track of things in this way might help people to change their [weight, steps, diet, blood glucose]?
   - How useful are you finding this for changing your [weight, steps, diet, blood glucose]?
2. Did the programme give you any **feedback** so far on changes to your [weight, steps, diet, blood glucose]?
   - How useful did you find it to have that feedback?
   - How do you think getting that feedback helps people to change their [weight, steps, diet, blood glucose]?
3. Overall, how useful have you found the online ‘Healthy Living’ programme?

- What has been the most important aspect of the programme for you?
- What is the main thing in your life that changed for you as a direct result of using this online programme?
- Was there anything that you would have liked to have seen included? If yes, why?
  - Initial demonstration of how to use the programme
  - Interaction with healthcare professional
    - How? (e.g. phone call support)
  - Moderated forum (peer support)
- What are your views on:
  - Content
    - Quizzes
    - Videos
  - Email communication (in terms of how many emails they received to prompt them to use the website)
  - Presentation (way it looked)
  - Tone of written articles (was the language easy to understand)
- How does Healthy Living compare to any other diabetes education programmes you may have attended, whether face-to-face (e.g. DESMOND) or digital (e.g. apps or website)?
- Would you recommend this programme to others?

1. Is there anything else about the Healthy Living programme we haven’t talked about yet that you’d like to tell me about before we finish?

*Thank you very much for taking the time to take part in our research.*
