# Supplementary material for: Service Users’ Experiences of a Nationwide Digital Type 2 Diabetes Self-Management Intervention (Healthy Living): Qualitative Interview Study
Source: JMIR Diabetes. 2024 Jul 18;9:e56276. doi: 10.2196/56276 (PMC11294771; doi:10.2196/56276)
Supplement: Multimedia Appendix 6 [file diabetes_v9i1e56276_app6.docx]

**Multimedia Appendix 6 – Coding Framework**

**Aim:** To explore participants’ experiences of using the ‘Healthy Living with type 2 diabetes’ programme.

**Specific objectives:**

- To understand how acceptable participants found different components of Healthy Living.
- To understand what content of Healthy Living participants engaged with.
- To understand any barriers to engagement with, and use of, Healthy Living.
- In line with the NIH-BCC framework, to investigate how Healthy Living is understood (‘intervention receipt’), and how this impacts on usage of intervention materials (‘intervention enactment’).

**GENERAL EXPERIENCES**

| **Code *(description)*** | | | | |
| --- | --- | --- | --- | --- |
| Journey before Healthy Living  *Experiences with GP and healthcare, hopes of Healthy Living, what patients wanted from programme* | Access and engagement with programme  *How often, use over time, how much of the Learn journey they completed, what device, how programme will be used going forward* | Positive comments on overall programme  *Navigation, usability, tone, presentation, trustworthiness, reasons for liking, most important feature of programme* | Negative comments on overall programme  *Navigation, usability, tone, presentation, reasons for disliking* | Use of other apps /programmes /websites outside of Healthy Living  *Comparison of Healthy Living to other programmes (e.g. DESMOND, X-PERT), use of Healthy Living in conjunction with other apps, information sought from other websites/resources* |
| **Sub-codes** | | | | |
| Other health conditions |  |  | Technical issues  Suggested improvements to programme |  |

**EDUCATIONAL CONTENT**

| **Code *(description)*** | | | | | |
| --- | --- | --- | --- | --- | --- |
| Positive comments on Learn Journey  *Reasons for liking, format, usability, structured vs. unstructured, how useful* | Negative comments on Learn Journey  *Reasons for disliking, format, usability, structured vs. unstructured, how useful* | Engagement with educational content  *What content, how often, use over time, how content is engaged with, motivation to continue through Learn Journey, what kept them engaged/not engaged, how Learn Journey will be used going forward* | Understanding of educational content  *Knowledge gained, how educational content is understood* | Enactment of educational content  *Behaviour change as a result of educational content (e.g. changes to diet, activity)* | Positive/negative comments on ‘Find Answers’ section  *Reasons for liking/disliking, format, usability, navigation* |
| **Sub-codes** | | | | | |
|  | Suggested improvements to Learn Journey |  |  |  | Suggested improvements to Find Answers |

**SELF-MANAGEMENT CONTENT**

| **Code *(description)*** | | | | |
| --- | --- | --- | --- | --- |
| Positive comments on self-management content  *Reasons for liking, how useful* | Negative comments on self-management content  *Reasons for disliking, how useful* | Engagement with self-management content  *What content, how often, use over time* | Understanding of self-management content  *Knowledge gained, how self-management content is understood* | Enactment of self-management content  *Behaviour change as a result of reading self-management content (e.g. management of moods [emotional], checking blood levels [medical])* |
| **Sub-codes** | | | | |
|  |  | Lack of recall of self-management content |  |  |

**BEHAVIOUR CHANGE TECHNIQUE (BCT) CONTENT**

| **Code *(description)*** | | | | | |
| --- | --- | --- | --- | --- | --- |
| Use of BCTs in Healthy Living  *What BCTs, how often, use over time* | Use of BCTs outside of Healthy Living  *E.g. via other apps, paper diaries, in their heads, etc.* | Understanding of BCTs  *How a BCT works in changing health behaviours* | Enactment of BCTs  *Behaviour change as a result of using the BCT* | Positive comments on BCT Tools  *Reasons for liking, format, usability, navigation, usefulness* | Negative comments on BCT Tools  *Reasons for disliking, format, usability, navigation, usefulness* |
| **Sub-codes** | | | | | |
| Lack of recall of BCT |  |  |  |  | Suggested improvements to BCT Tools |

**SUPPORT**

| **Code *(description)*** | | | | |
| --- | --- | --- | --- | --- |
| Positive comments on support provided throughout programme  *Reasons for liking, how support has been accessed* | Negative comments on support provided throughout programme  *Reasons for disliking, how support has been accessed* | Facilitated access  *Thoughts on (lack of) facilitated access, how facilitated access would/would not meet needs* | Online forum  *Thoughts on (lack of) online forum* | Support sought as a result of programme  *How programme has been used to access further support* |
| **Sub-codes** | | | | |
| ‘People’s stories’ videos (positive)  Email content received (positive) *(e.g. content, frequency, tone)*  Self-assessment quizzes (positive)  Technical support *(e.g. for use of website)* | ‘People’s stories’ videos (negative)  Email content received (negative) *(e.g. content, frequency, tone)*  Lack of recall of email content received  Self-assessment quizzes (negative)  Suggested improvements to support provided on the programme |  | How an online forum would meet needs  How an online forum would not meet needs  Feeling of belonging to a community (or lack of) | Signposting  Conversations with health professionals outside the programme |
