# Supplementary material for: Service Users’ Experiences of a Nationwide Digital Type 2 Diabetes Self-Management Intervention (Healthy Living): Qualitative Interview Study
Source: JMIR Diabetes. 2024 Jul 18;9:e56276. doi: 10.2196/56276 (PMC11294771; doi:10.2196/56276)
Supplement: Multimedia Appendix 7 [file diabetes_v9i1e56276_app7.docx]

**IMPORTANCE OF TECHNOLOGICAL, PROFESSIONAL AND SOCIAL INTERACTIVITY**

**Online forum only useful if moderated by credible HCPs**

**Videos with ‘people’s stories’ provided a form of support that some benefitted from**

**Desire for existing tools to link up with Healthy Living**

**INTERACTION WITH HEALTHCARE PROFESSIONALS**

**INTERACTION WITH OTHER PEOPLE LIVING WITH T2DM**

**Participants suggested ways to increase interaction with HCPs on the programme**

**Programme particularly suited to newly diagnosed patients**

**Learned new information about medical management**

**Wanted more information about dietary management**

**Healthy Living provided participants with a trusted source of information**

**Some did not encounter the emotional management content if not progressed far enough through the programme**

**Desire for more interactivity/ prompts from Healthy Living**

**Participants already used existing methods to self-monitor**

**Content prompted some to book appointment with GP about their low mood**

**Emotional management content was useful**

**Emotional management content was unexpected**

**Some disengaged when they encountered a topic not relevant to them**

**But some wanted to be able to select topics of their choice instead**

**Liked that information was presented in modules**

**INTERACTION WITH OTHER APPS AND DEVICES**

**EXPERIENCES OF STRUCTURED EDUCATION**

**“INFORMATION’S THERE AT THE TOUCH OF A BUTTON”**

**IMPROVED EMOTIONAL MANAGEMENT**

**PARTICIPANTS’ EXPERIENCES OF USING HEALTHY LIVING**
